# Wild mouse gut microbiota limits initial tuberculosis infection in BALB/c mice

**DOI:** 10.1371/journal.pone.0288290

**Published:** 2023-07-26

**Authors:** Min Xie, Chen-Yu Tsai, Zachary L. McAdams, Myo Oo, Mark Hansen, Maureen Dougher, Alexander Sansano, Anderson Watson, Katherine LoMauro, Rosleine Antilus-Sainte, Aaron Ericsson, Véronique Dartois, Martin Gengenbacher

**Affiliations:** 1 Center for Discovery and Innovation, Hackensack Meridian Health, Nutley, New Jersey, United States of America; 2 Molecular Pathogenesis and Therapeutics Program, University of Missouri, Columbia, Missouri, United States of America; 3 University of Missouri Metagenomics Center, Department of Veterinary Pathobiology, University of Missouri, Columbia, Missouri, United States of America; 4 Hackensack Meridian School of Medicine, Nutley, New Jersey, United States of America; Centenary Institute, AUSTRALIA

## Abstract

Mouse models are critical tools in tuberculosis (TB) research. Recent studies have demonstrated that the wild mouse gut microbiota promotes host fitness and improves disease resistance. Here we examine whether the wild mouse gut microbiota alters the immunopathology of TB in BALB/c mice. Conventional BALB/c mice (LabC) and mice born to germ-free BALB/c mothers reconstituted with the wild mouse gut microbiota (WildR) were used in our studies. WildR mice controlled initial TB infection better than LabC mice. The microbial gut communities of LabC mice and WildR mice had similar richness but significantly different composition prior to infection. TB reduced the gut community richness in both cohorts while differences in community composition remained indicating a general TB-induced dysbiosis. The wild mouse gut microbiota did not alter the typical lung histopathology of TB in the BALB/c model that includes unstructured immune cell infiltrates with infected foamy macrophages invading alveolar spaces. Animals of both cohorts mounted robust T cell responses in lungs and spleen with lower absolute counts of CD4 and CD8 T cells in lungs of WildR mice during acute infection, corresponding with observed differences in pathogen load. In summary, LabC mice and WildR mice showed largely overlapping TB immunopathology and pathogen kinetics, with WildR mice controlling early acute infection better than LabC mice.

## Introduction

Tuberculosis (TB) remains a major threat to global public health and years of progress made towards ending TB have been reversed by the COVID19 pandemic [1]. Predictive preclinical models for testing novel drugs and vaccine candidates are required to achieve the targets set forth in the End TB Strategy.

Laboratory mice have played a critical role in TB preclinical therapy development and studies of *Mycobacterium tuberculosis* (Mtb) immunopathology. However, they do not always align closely with clinical observations [2, 3]. Most of the common mouse strains used in research have been inbred for many decades under specific pathogen-free conditions in a controlled environment without dietary variations that led to a loss in microbiota diversity and richness [4]. Moreover, laboratory animals are not exposed to microbes that wild-living mice constantly encounter. Several studies have highlighted that exposure to microbes and pathogens, including mites and pinworms, and the presence of the wild mouse microbiota may improve the translational value of murine models and better reflect human immune responses [4–8]. However, introduction of murine pathogens to research poses significant challenges for laboratory animal facilities and is often prohibitive because such pathogens are notoriously difficult to contain [9]. Rosshart *et al*. have defined the wild mouse gut microbiota, which was significantly more diverse and various species were differentially represented as compared to conventional laboratory mice [4]. Reconstitution of germ-free laboratory mice with the wild-mouse gut microbiota enabled their offspring, termed “WildR” by vertical transfer, to experience microbiota-mediated effects before and after birth [10, 11]. The gut microbiota of WildR mice was stable over several generations, promoted host fitness and improved disease resistance to influenza virus infection and mutagen/inflammation-induced colorectal tumorigenesis [4]. A recent study showed that previous intestinal infections in WildR mice increase the gut microbiota’s resistance to subsequent infections by inhibiting pathogen respiration [12]. Taken altogether, WildR mice have demonstrated translational value in several research fields owing to their wild mouse gut microbiota.

The onset and recurrence of active TB infection in humans are characterized by a decline in the diversity of the gut microbiota [13, 14]. Similarly, mice experience a rapid loss of fecal microbiota diversity upon Mtb infection [15]. Depletion of the microbiome by broad-spectrum antibiotics rendered mice more susceptible to TB and restoring the gut microbiota by fecal transfer reduced the bacterial load of Mtb [16]. Chemotherapy with first-line antimycobacterials led to dysbiosis that persisted beyond the end of drug treatment for at least 3 months in Mtb-infected mice and at least 1.2 years in TB patients [17, 18]. Taken altogether, the crosstalk through the gut-lung axis is bidirectional and impacts both Mtb in the lung and the microbial community of the gut. Because there are striking differences between the microbiota of laboratory mice and wild-living mice, we examine whether laboratory mice reconstituted with the wild-mouse gut microbiome may better resemble the hallmarks of clinical TB than conventional laboratory mice. We chose the BALB/c mouse as the host organism for our studies as this strain is widely used in TB preclinical development. Here we investigate the impact of the wild-mouse gut microbiota on TB infection in the BALB/c model using organ bacterial burden, lung histopathology, microbiome changes, cytokine profiles, and T cell responses as readouts.

## Materials and methods

### Ethics statement

This study was carried out in strict accordance with the recommendations in the Guide for the Care and Use of Laboratory Animals of the National Institutes of Health. The protocol was approved by the Institutional Animal Care and Use Committee of Hackensack Meridian Health (Protocol Number: 265) and all efforts were made to minimize suffering and pain.

### Bacterial strains and media

Mtb H37Rv (ATCC #27294) was cultured in Middlebrook 7H9 broth supplemented with 0.05% Tween-80, 0.2% glycerol and 10% albumin-dextrose-catalase enrichment (Becton Dickinson) at 37°C and 80 rpm or on Middlebrook 7H11 agar containing 0.5% glycerol and 10% oleic-acid-albumin-dextrose-catalase enrichment (Becton Dickinson) at 37°C. Colonies were counted after 3–4 weeks of incubation.

### Aerosol infection of mice and pathogen load determination

Cohorts of 8–9 weeks old female BALB/c mice born to germ-free BALB/c mothers reconstituted with the gut microbiota of wild-living mice, termed WildR herein, and age-matched conventional laboratory BALB/c females, termed LabC, were obtained from Taconic and maintained under sterile conditions using individually ventilated cages. Both cohorts were simultaneously infected while maintaining strict separation. The infection dose of 100–200 colony-forming units (CFU) of aerosolized Mtb H37Rv was delivered by a whole-body inhalation exposure system (Glas-Col) and verified 3 h post-infection [15]. The bacterial loads of lungs and spleens were determined by plating serial dilutions of individual organs homogenized in 1 ml phosphate buffered saline (PBS)/0.05% Tween 80 onto Middlebrook 7H11 agar.

### Feces collection, DNA extraction, 16S rRNA library preparation and sequencing

Up to 2 pieces of feces from individual mice were sterilely collected, heat inactivated and subjected to bead-beating using Lysing Matrix B, 0.1 mm silica spheres (MP Biomedicals). DNA was extracted using a column-based DNA stool mini kit (Qiagen) following the manufacturer’s protocol. Final DNA concentrations and quality were determined by fluorometric analysis (Qubit 2.0 Invitrogen) using quant-iT BR dsDNA reagent kits (Invitrogen) and normalized to a uniform concentration and volume. Microbial 16S rRNA amplicons were constructed by amplification of the V4 region of the 16S rRNA gene with universal primers previously developed against the V4 region, flanked by Illumina standard adapter sequences [19, 20]. Dual-indexed forward and reverse primers were used in all reactions. Amplicon pools at equal concentrations were combined, thoroughly mixed, and purified using Axyprep MagPCR clean-up beads (Axygen) prior to final analysis on a fragment analyzer automated electrophoresis system (Advanced Analytical). Amplicon pools were then diluted following Illumina’s standard protocol for sequencing on a MiSeq instrument generating 2x250 bp paired-end reads.

### Informatics analysis

16S rRNA amplicon sequences were processed using Quantitative Insights into Microbial Ecology 2 (QIIME2) [21] v2021.8. Briefly, forward and reverse reads were timed of the universal (515F/806R) primers using *cutadapt* [22]. If found, the reverse complement of the primer to the reverse read was then removed from the forward reads as were all bases downstream. Thus, a forward read could be trimmed at both ends if the insert was shorter than the amplicon length. The same approach was taken on reverse read, but with the primer ins in the opposite role. Read pairs were rejected if one read or the other did not match a 5’ primer, and an error rate of 0.1 was allowed. Two passes were made over each read to ensure the removal of the second primer. A minimal overlap of 3 bases with the 3’ end of the primer sequence was required for removal.

DADA2 (*q2-dada2*) was used to denoise, de-replicate, and count amplicon sequence variants (ASVs) incorporating the following parameters: (i) forward and reverse reads were truncated to 150 bases; (ii) forward and reverse reads with the number of expected errors higher than 2.0 were discarded; and (iii) chimeras were detected using the “consensus” method and removed. ASVs were filtered to between 249 and 257 bp in length. Taxonomies were assigned to final sequences using the Silva V138 16S (515F/806R) rRNA SSU reference database [23] using the classify-sklearn procedure.

Microbiome data were analyzed using the open-source R statistical software v4.2.2 [24]. The DADA2-generated feature table was rarefied to 87,953 total features per sample using the *rrarefy* function from the *vegan* library [25, 26]. Community richness (i.e., number of unique taxa within a community) was determined using the *microbiome* library [27]. To assess differences in community composition between samples, a distance matrix was first generated from a quarter-root-transformed feature table with Bray-Curtis distance metrics. A principal coordinate analysis of the distance matrix was then performed using the *pcoa* function from the *ape* [28] library with a Calliez correction. Statistical differences in community composition were determined using the *adonis2* function from the *vegan* library [25, 26] which performs a two-way permutational analysis of variance (PERMANOVA). Differential abundance testing was performed using ALDEx2 [29] with the recommended 128 Monte Carlo simulations. Differentially abundant features were determined based on a Benjamini-Hochberg corrected Wilcoxon Rank-Sum test *p* value < 0.05.

### Bacterial load of organs and histopathology analysis

Organs of euthanized animals were aseptically collected. Half of the spleen and the left lung lobe were homogenized individually in gentleMACS M tubes (Miltenyi) with 1 ml PBS/0.05% Tween 80 using a gentleMACS Octo Dissociator (Miltenyi). Subsequently, serial dilutions of organ homogenates were plated on Middlebrook 7H11 agar. For histopathology analysis entire lungs of two mice were perfused with 10 ml PBS and fixed overnight in 10% neutral buffered formalin. Organs were processed, embedded in paraffin, and sectioned at 3 μm thickness with 50 μm between pairs of two adjacent sections throughout the organ. Each pair of sections was subjected to hematoxylin and eosin (H&E) staining and to Ziehl-Neelsen staining, respectively. Histology slides were imaged using a 3D HiStech slide scanner (Pannoramic Desk) to quantify the number of immune cell infiltrates per slide. Images of pathology details were taken using a NiE Research Microscope equipped with CFI Plan APO Lambda objectives and NIS-Elements AR Software (Nikon Instruments).

### Multiplex cytokine analysis of organ homogenates

Organ homogenates were spun at 14,000 rpm for 10 min at 4°C in a microcentrifuge and the supernatants were sterilized by passing them twice through 0.22 μm Spin-X Centrifuge Tube Filters (Corning). For cytokine profiling, we utilized the Bio-Plex Pro Mouse Cytokine 23-plex mouse cytokine assay (BioRAD) according to the manufacturer’s protocol and the FLEXMAP 3D system (Luminex) for data acquisition. The instrument was calibrated, and performance was verified using the FLEXMAP 3D Calibration Kit and the FLEXMAP 3D Performance Verification Kit (Luminex) following the manufacturer’s instructions. Raw data was analyzed using the Belysa Immunoassay Curve-Fitting software V1.0.19 (Merck) and the GraphPad Prism 11 software was used to visualize cytokine profiles and to perform statistical analysis. Principal component analysis was performed in R v.4.2.0 (2022-04-22) with Platform: aarch64-apple-darwin20 (64-bit). The following R packages were used: dplyr (v.1.0.9), ggplot2 (v.3.3.6) [30, 31].

### Preparation of single cell suspension, cell staining and flow cytometry analysis

The superior-, middle-, inferior- and postcaval-lung lobes were dissociated with 2.5 ml of Roswell Park Memorial Institute 1640 Medium (RPMI)/53 μg liberase (Roche) in C tubes using a gentleMACS Octo Dissociator (Miltenyi). The tissue suspension was then incubated for 1 h at 37°C and single cells were counted using an automatic instrument (Lunar II). For cell surface staining, ~2 million cells were washed and resuspended in FACS buffer (PBS, 1% bovine serum albumin, 0.5% sodium azide). Cells were first incubated with Live/Dead Fixable Near-IR (Thermo Fisher Scientific). After washing, cells were incubated for 30 min with a cocktail of fluorochrome-conjugated specific antibodies at appropriate concentrations: anti-mouse CD45 Alexa Fluor 700 (clone 30-F11, Biolegend), anti-mouse CD3 BV605 (clone 17A2, Biolegend), anti-mouse CD4 BUV395 (clone GK1.5, BD), anti-mouse CD8a BB700 (clone 53–6.7, BD), anti-mouse γδTCR FITC (clone GL3, Biolegend) and anti-mouse B220 BUV496 (clone RA3-6B2, BD). Cells were then washed, fixed for 60 min with Cytofix/Cytoperm Fixation and Permeabilization Solution (BD Biosciences) and then acquired on a FACSymphony A3 Cell Analyzer (BD Biosciences). Data analysis was performed using the FlowJo Software V10.8.1 (BD Biosciences).

## Results

### WildR mice control initial acute TB lung infection better than conventional mice

**Fig 1:**
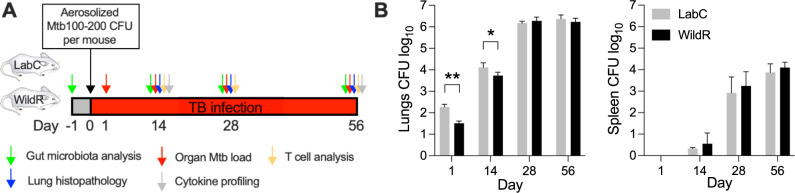
WildR mice control acute pulmonary TB infection better than LabC mice. BALB/c mice with conventional laboratory mouse gut microbiome (LabC) and BALB/c mice born to germ-free mothers reconstituted with wild-mouse gut microbiota (WildR) were infected with a low dose of aerosolized Mtb H37Rv. **A.** Study design and readouts. **B.** At designated time points, half of the organs of 6 mice per cohort were homogenized and plated on agar to determine the pathogen load in lungs (left panel) and spleen (right panel) over time. Mean and SD are shown. Data of individual time points were analyzed using the two-sided Student’s *t*-test. *p<0.05, **p<0.001.

We used commercially available age-matched cohorts of LabC mice and WildR mice [10, 11] that were generated in the BALB/c background. To study the impact of wild-mouse gut microbiota on TB, we simultaneously infected both cohorts in the same aerosol generator chamber by a low dose of aerosolized Mtb. Strict separation between the two animal groups was maintained during the infection process and throughout the experiment to prevent microbiome transfer. We verified delivery of the initial inoculum dose at 24 h post-infection and monitored the progress of TB thereafter by determining the pathogen load of organs at 14, 28 and 56 days. The study design is depicted in Fig 1A. The course of low dose Mtb infection in mice can be divided into the acute phase characterized by rapid Mtb growth (until ~4 weeks), and the chronic phase during which a high pathogen load in organs is maintained [32]. The lungs of WildR mice at 1 day and 14 days post-infection had a significantly lower bacterial burden than LabC mice, and this difference dissipated thereafter (Fig 1B left). In contrast, animals of both cohorts had a similar pathogen load in the spleen at individual time points (Fig 1B right). This suggests that the gut microbiota may shape innate immunity leading to different degrees of TB control in WildR mice and LabC mice in the early time points following infection. Dissemination of TB to secondary organs such as the spleen was not affected by the gut microbiota.

### TB infection affects the composition of the LabC and WildR gut microbiome

The conventional BALB/c mouse model is widely used in TB research, including preclinical development of novel therapies. Laboratory mice have been bred under specific pathogen-free conditions for many generations which led to alteration of their gut microbiota, and consequently, changes in their immune responses relative to wild living mice [8]. It has been reported that TB infection has a profound impact on the gut microbiota of humans and mice [13, 14]. To investigate whether the gut microbiota impacts the course of TB in the BALB/c model, we used animals born to germ-free mothers that were inoculated with the gut microbiota of wild-living mice, and age-matched conventional BALB/c mice. Fecal samples obtained from individual mice of both cohorts prior to and during TB infection were subjected to DNA isolation and sequencing of the 16S rRNA gene V4 region. DADA2 denoising of paired-end reads identified an average of 102,631 (± 7,160) amplicon sequence variants (ASVs) per sample (S1 Table).

**Fig 2:**
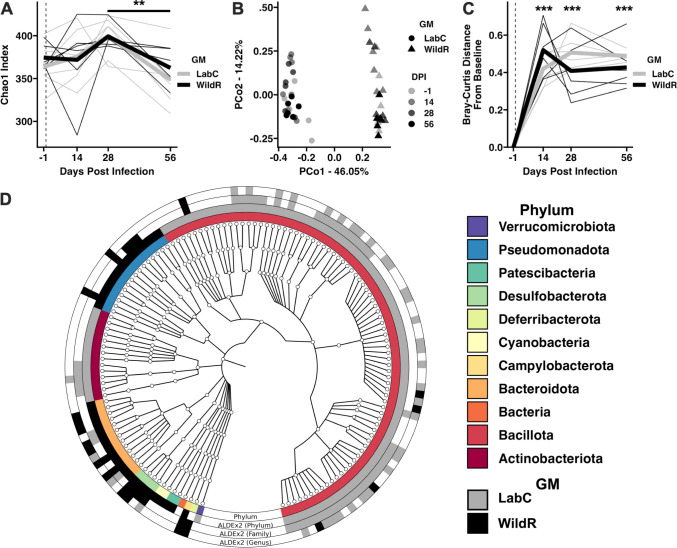
Microbial community analysis of LabC and WildR microbiomes prior to and during TB infection. **A.** Line plot depicting within sample richness (Chao1 Index) across each timepoint. Thin lines represent individual mice. Thick lines represent average richness of each GMs. ** *p <* 0.01 D28 v. D56 Tukey *post-hoc* test. Dashed line indicates D0 (day of infection). **B.** Principal coordinate analysis using Bray-Curtis distances depicting significant differences in microbial composition between GMs (F = 40.39, *p <* 0.001) and across time (F = 2.47, *p* = 0.045), GM:Time (F = 2.29, *p* = 0.053). Two-way PERMANOVA. **C.** Line plot depicting Bray-Curtis distances from baseline (D-1). Thin lines represent individual mice. Thick lines represent average richness for both GMs. *** *p* < 0.001 Tukey *post-hoc* comparison to D-1. Dashed line indicates D0 (day of infection). **D.** Phylogenetic tree depicting ALDEx2-determined differentially abundant taxa (BH-corrected *p* < 0.05) between GMs. From innermost ring out: First ring depicts phylum, second differentially abundant phyla, third differentially abundant families, fourth differentially abundant genera.

Microbial community analysis of LabC and WildR gut microbiomes revealed that the two communities were similar in community richness prior to and during acute TB infection but were both reduced during chronic infection (Fig 2A). LabC and WildR communities significantly differed in microbial composition prior to and during TB infection suggesting that TB infection induced a dysbiosis of the gut microbiome regardless of the initial community structure (Fig 2B). In support of this, we assessed the Bray-Curtis dissimilarity of samples collected during TB infection compared to pre-infection samples. Infection with TB significantly increased the Bray-Curtis dissimilarity of microbial community relative to pre-infection samples within the first 14 days of infection and persisted through day 56 (Fig 2C). This TB-induced dysbiosis of the gut microbiome has been previously reported in both human and mouse TB infections (reviewed in [33]).

Given the significant differences in overall community composition between LabC and WildR mice (Fig 2B), we next applied ALDEx2 [29] to detect differentially abundant taxa at the phylum, family, and genus levels (Fig 2D). At the phylum level, *Bacteroidota* (formerly *Bacteroidetes*) and *Pseudomonadota* (formerly *Proteobacteria*) were dominant in WildR mice, whereas *Bacillota* (formerly *Firmicutes*) and *Actinobacteriodota* were enriched in the LabC mice. *Verrucomicrobiota* and *Campylobacterota* were only detected in LabC and WildR mice, respectively. Members of the *Bacillota* phylum including *Ruminococcus* (Family *Ruminococcaceae*), *Butyricicoccus* (Family *Butyricicoccaceae*), and multiple *Lachnospiraceae* groups were enriched in LabC mice. LabC mice also exhibited a higher proportion of *Roseburia* compared to WildR mice. Interestingly, *Roseburia* are butyrate-producing bacteria that are associated with beneficial health outcomes in mice and in humans [34]. WildR mice were enriched for *Odoribacter* (Family *Marinifilaceae*), *Helicobacter* (Family *Helicobacteraceae*), and genera within the *Rikenellaceae* family. The sulfur-reducing genera *Bilophila* and *Desulfovibrio* were also differentially abundant in WildR mice. A full list of differentially abundant taxa at the phylum, family, and genus levels is provided in S2 Table. Our findings in the BALB/c WildR model are consistent with the C57BL/6 WildR mice [4].

### LabC mice and WildR mice develop comparable pulmonary TB histopathology

The gut microbiota of WildR mice was shown to promote host fitness and improve resistance to influenza infection [4]. To examine whether the microbiome impacts the lung histopathology of TB, we compared H&E-stained lung sections of LabC mice and WildR mice at 14-, 28- and 56-days post-infection. Typical TB lung lesions in conventional BALB/c mice are cellular and consist of lymphocyte aggregates, epithelioid histocytes and intra-alveolar foamy macrophages [35–37].

**Fig 3:**
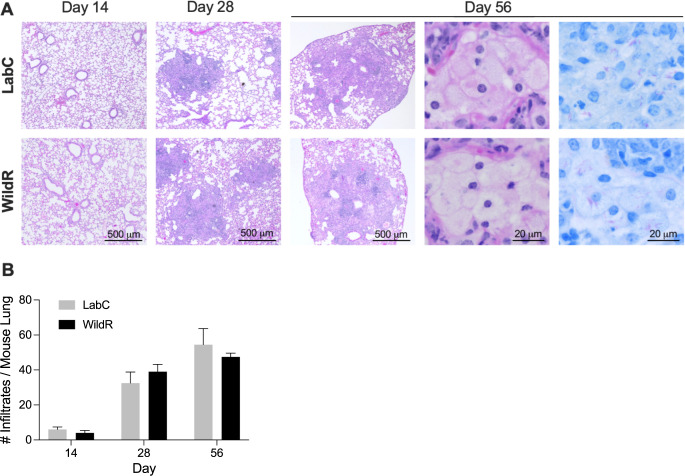
Pulmonary histopathology of TB is independent of the intestinal microbiome. **A.** At 14-, 28- and 56-days post-infection lungs of animals were formalin-fixed, paraffin-embedded, sectioned at 3 μm, mounted on glass slides, stained with hematoxylin & eosin (eight panels on the left) or Ziehl-Neelsen (two panels on the right) and examined by brightfield microscopy. Acid-fast positive bacilli are shown as purple rods. Representative areas of LabC mice and WildR mice are shown. **B.** All lung lobes of two mice per cohort were completely sectioned with 50 μm steps between individual sections. Pulmonary infiltrates with a diameter of >100 μm were quantified. Infiltrates present in multiple sections were only counted once. Data are shown as mean and SD. The unpaired student’s *t*-test was used to perform pairwise comparisons at designated time points. Sections of all lung lobes at designated time points are shown in S2 Fig.

At 14 days post-infection lymphocytes had accumulated in the lungs of LabC mice and WildR mice, mostly around larger airways (Fig 3A). At 28 days post-infection, unstructured cellular lesions were present, with a diameter often exceeding 0.5 mm that further increased until the end of the study at 56 days (Fig 3A). Foamy macrophages appearing more transparent due to their accumulation of lipid bodies filled out many alveolar spaces and were infected with acid-fast bacilli (Fig 3A, two right panels). The representation of TB lung pathology in both cohorts was very similar (Fig 3A, top and bottom row) and closely resembled reports in conventional BALB/c mice [35–37]. We then quantified the number of pulmonary immune cell infiltrates in H&E-stained sections. As expected, the number of TB lesions increased over the course of infection reaching 45 to 55 infiltrates per animal at the end of the study with no significant difference between the two cohorts (Fig 3B). We conclude that the wild mouse gut microbiome does not impact the number of lung lesions and the lesion organization in the BALB/c model.

### WildR mice have a less pronounced T cell response during acute TB infection

**Fig 4:**
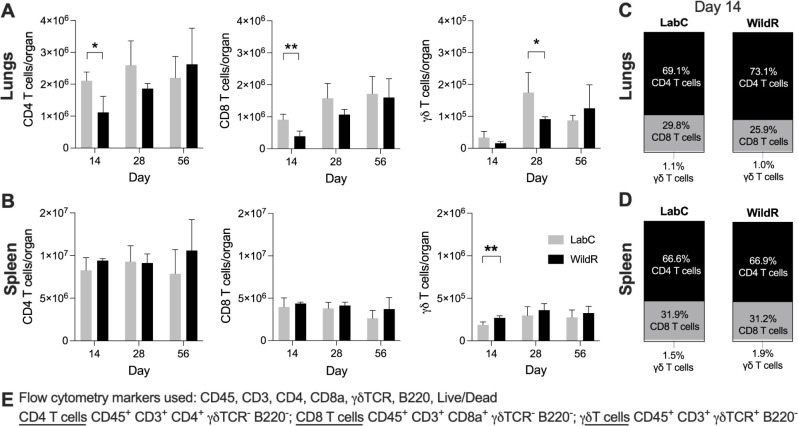
WildR mice have a less pronounced T cell response in the lungs during acute TB infection. At designated time points after Mtb infection, groups of four mice were used to prepare organ single-cell suspensions for flow cytometry analysis of T cell subsets. Absolute numbers of CD4, CD8 and γδT cells in lungs (**A**) and in the spleen (**B**), and frequencies of CD4, CD8 and γδT cells among all T cells in lungs (**C**) and in the spleen (**D**). Data are shown as mean and SD. Unpaired Student’s *t*-tests were performed for pairwise analysis of T cell subsets of four LabC mice and four WildR mice at 14-, 28- and 56-days post-infection. *p<0.05, **p<0.001. **E.** Flow cytometry markers used in this study and definitions of analyzed T cell subsets.

T cells play an essential role in controlling TB infection [38] and growing evidence suggest that T-cell responses can be shaped by the microbiota [39]. To determine the impact of the gut microbiota on T cell immunity to TB, we measured the dynamics of CD4, CD8 and γδ T cells during acute and chronic infection. WildR and LabC mice showed a robust expansion of pulmonary and splenic T cell subsets in response to TB infection (Fig 4A and 4B), which is consistent with the literature [40]. At 14 days post-infection, lungs of WildR mice had lower absolute counts of CD4 and CD8 T cells than LabC mice and these differences faded to undetectable levels at later time points (Fig 4A). Similarly, WildR mice has slightly less γδ T-cells in the lungs at 28 days (Fig 4A). In the spleen, CD4 and CD8 T cell counts were on par throughout the experiment (Fig 4B). In addition, lungs of WildR mice had slightly more γδ T-cells than LabC mice at days 14 post-infection (Fig 4B). CD4 and CD8 T cells represented the majority of T-cell populations in WildR and LabC mice during infection (Fig 4C). WildR mice generally had a less pronounced T-cell response in the lungs at early time points, compared to LabC mice, and a comparable T-cell response in the spleen throughout the TB infections.

**Fig 5:**
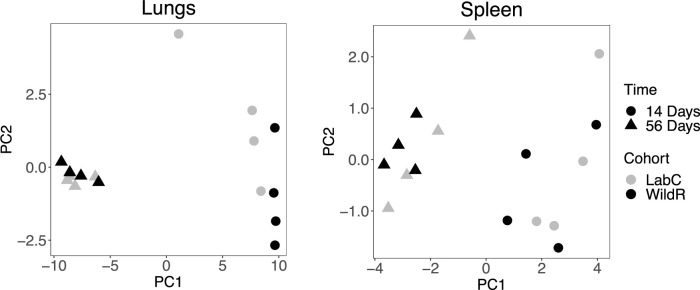
The wild-mouse gut microbiome does not affect cytokine profiles of Mtb-infected organs. Principal component analysis of protein levels of 23 cytokines in the lungs (left) and spleen (right) for four LabC mice (grey) and four WildR mice (black) at 14- (circle) and 56-days (triangle) post-infection. Each dot represents the organ cytokine profile of one mouse. Concentrations of individual cytokines are shown in S1 Fig.

We also examined the protein levels of 23 cytokines and chemokines in the lungs and spleen of LabC and WildR mice at the acute and chronic phases of TB infection. Most cytokine and chemokine levels were much higher in the lungs than in the spleens (S1 Fig). We thus performed principal component analysis to explore if different gut microbiomes would affect cytokine expressions in response to TB infection (Fig 5). Cytokine profiles of mice from the same time points tend to cluster together regardless of their gut microbiome. This suggested that measured cytokine profiles during TB infection are independent of the gut microbiota. In summary, our data shows that the fecal microbiome impacts early T cell responses during acute infection which did not translate into changes in the cytokine profiles.

## Discussion

The BALB/c mouse model has been a workhorse in TB preclinical therapy development for many decades, but results do not always align with humans. Mice having the gut microbiota of their wild-living relatives have better host fitness, improved disease resistance and might model humans more closely [4]. Here we tested whether introducing the wild mouse gut microbiota to the BALB/c host (WildR) would alter the representation and course of TB infection. Conventional BALB/c mice with the microbiome of continuously inbred animals (LabC) served as a reference. Consistent with previous reports, the compositions of the intestinal microbiomes of LabC and WildR mice had differently composed microbial gut communities (Fig 2D) [4]. The WildR mice were enrichen in *Bacteroidota* and *Pseudomonadota*, whereas *Bacillota* and *Actinobacteriodota* were enriched in the LabC mice. Both LabC mice and WildR mice had microbiome taxa that are not common in specific pathogen free research environments and might be specific to our animal supplier’s facility. *Helicobacter* and *Odoribacter* were enriched in WildR mice. Colonization of the murine gut with *Helicobacter hepaticus* was associated with increased Mtb growth, elevated inflammation through CD4 and CD8 T cell activation [41] and more sever pulmonary pathology [42]. In contrast, *Helicobacter pylori* infection was associated with immunoprotection against TB in macaques and in humans [43]. The presence of *Helicobacter* species (*H*. *bilis*, *H*. *ganmani*, *H*. *hepaticus*, *H*. *mastomyrinus*, *H*. *rodentium*, *H*. *typhlonius*) that are part of the WildR gut microbiota [4] were beneficial or had no impact on TB infection in our studies. TB infection caused a major perturbation of the intestinal microbiome of both cohorts and induced a significant loss in community richness during the chronic infection stage (Fig 2A). *Roseburia* species, were more abundant in LabC mice than in WildR mice. The higher abundance of *Roseburia* might be beneficial for mice during chronic TB infections [34]. Our data is consistent with previous reports indicating significant differences in the relative abundance of members of the *Lachospiraceae* family in response to TB infection [15].

LabC mice showed the typical biphasic course of pulmonary TB infection, including the acute phase until day 28 followed by the chronic phase [32]. While in principle WildR mice phenocopied the pathogen kinetics of LabC mice, the number of bacilli detected in lungs was lower at 1- and 14-days post-infection (Fig 1B). The observed difference in lungs did not impact dissemination to the spleen (Fig 1C). Both cohorts share the same genetic background, were age-matched and were infected simultaneously in one infection chamber while maintaining strict separation between microbiomes. Therefore, a comparable Mtb dose was likely deposited in lungs of all study animals. The lower lung bacterial load we observed at 1- and 14-days in WildR cohort might indicate a better early innate immune control in these mice, which quickly faded at later study time points (Fig 1B). The axis of innate immunity and the microbiome is an area of ongoing research [44–46]. Germ-free mice for instance have a profoundly altered innate immune system and the gut is a signal hub that integrates environmental inputs to affect immunity and the response to infection [47]. The communication of the intestinal microbiota and innate immunity is bidirectional [48]. In line with this, we find that Mtb triggers gut microbiota dysbiosis, and in turn the wild mouse microbiome may have impacted the early events of TB infection when innate immune responses dominate. Growing evidence suggests that TB susceptibility is impacted by the microbial community in the gut (reviewed in [49]) and our results further support this key finding.

The lower pathogen load in the lungs of WildR mice at 14 days post-infection did not translate into a measurable difference in pulmonary histopathology including the number of lesions and their organization and the absolute numbers of CD4 and CD8 T cells in our studies correlated with the organ bacterial load rather than the microbiome changes (Figs 3 and 4). The cytokine and chemokine profiles we measured in infected organs of LabC and WildR mice showed a large overlap (S1 Fig). A principal component analysis of data revealed a clear separation of animals by time points post-infection but not between microbiomes (Fig 5), demonstrating that cytokine and chemokine profiles during TB infection are independent of the gut microbiota. In a high-dose influenza A infection model using C57BL/6 mice, the wild mouse gut microbiota led to significant changes in cytokine profiles and was protective [4]. Such high-dose pathogen models typically assess immunity in a short time window after inoculation which was not the focus of our experiments. The nature of the infectious agent, the vastly different inoculation dose, the mouse strain and the choice of experimental endpoints may have contributed to the lack of cytokine profile changes in our studies.

In summary, TB infection in BALB/c mice causes dysbiosis of the gut microbiome regardless of the initial community structure. WildR mice control initial acute infection (low pathogen burden) better, possibly through enhanced immunity. With the onset of chronic TB characterized by a high pathogen load however, this advantage fades over the course of infection. Further studies are required to elucidate how the intestinal microbiome shapes immunity in the BALB/c model.

## Supporting information

S1 Fig The cytokine profile measured in BALB/c mice having TB is independent of the gut microbiota. Organ homogenates obtained at designated time points were subjected to cytokine and chemokine quantification using the multiplex technology. The data are shown as mean and SD. Paired Student’s *t*-tests were performed to analyze individual analytes of four LabC mice and four WildR mice at 14- and 56-days post-infection.
(PDF)

S2 Fig Pulmonary TB pathology of LabC mice and WildR. Lungs of animals infected for 14, 28 and 56 days were formalin fixed, paraffin-embedded, sectioned and stained with hematoxylin and eosin. Scale bar, 0.5 cm.
(PDF)

S1 Table Read counts of the 16S rRNA V4 region for individual feces samples. (PDF)

S2 Table Differentially abundant taxa at the phylum, family, and genus levels. (XLSX)
