# Supplementary material for: Wild mouse gut microbiota limits initial tuberculosis infection in BALB/c mice
Source: PLoS One. 2023 Jul 26;18(7):e0288290. doi: 10.1371/journal.pone.0288290 (PMC10370681; doi:10.1371/journal.pone.0288290)
Supplement: S1 Fig — Organ homogenates obtained at designated time points were subjected to cytokine and chemokine quantification using the multiplex technology. The data are shown as mean and SD. Paired Student’s t-tests were performed to analyze individual analytes of four LabC mice and four WildR mice at 14- and 56-days post-infection. (PDF) [file pone.0288290.s001.pdf]

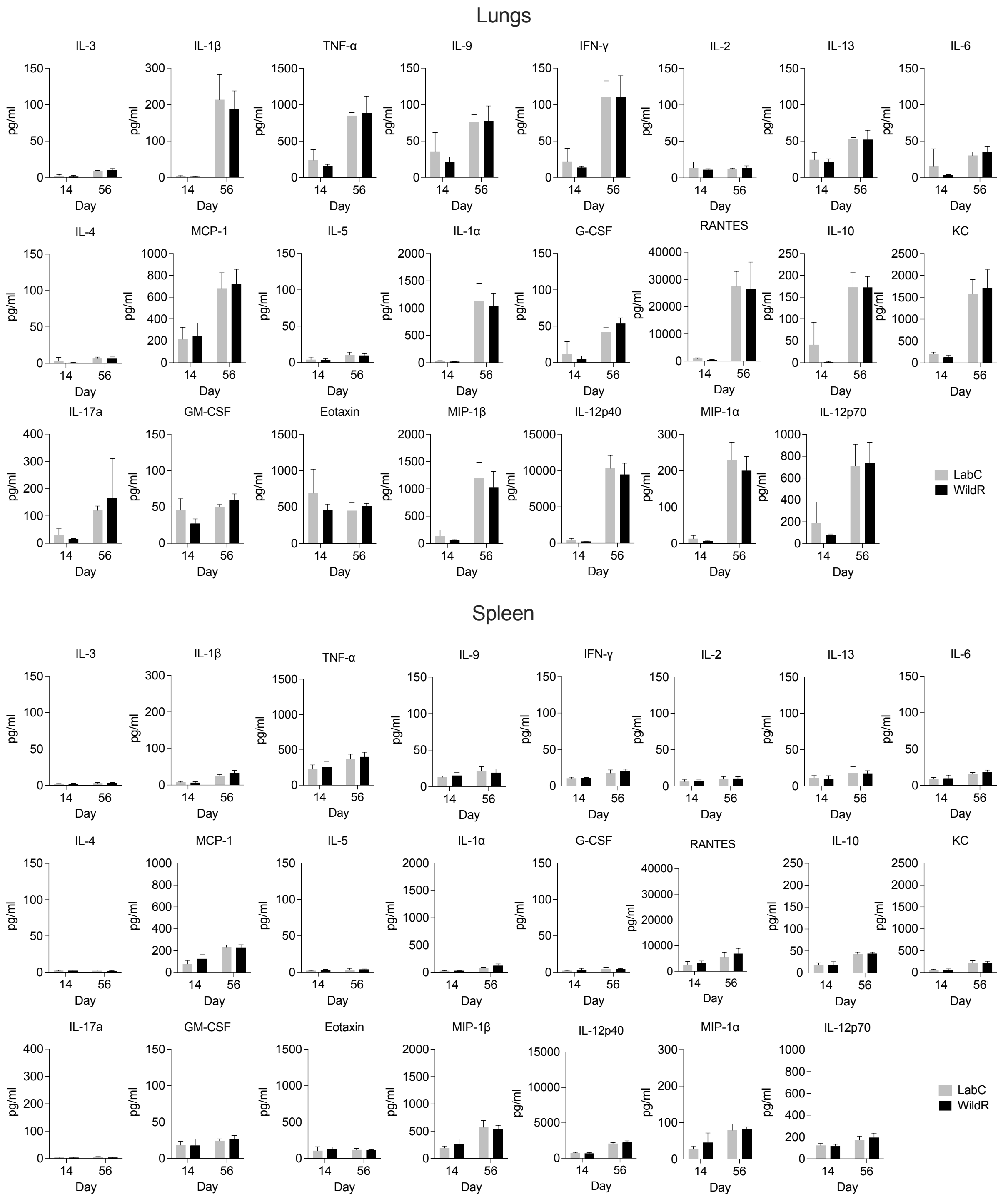


**S1 Figure. The cytokine profile measured in BALB/c mice having TB is independent of the gut microbiota.** Organ homogenates obtained at designated time points were subjected to cytokine and chemokine quantification using the multiplex technology. The data are shown as mean and SD. Paired Student's *t*-tests were performed to analyze individual analytes of four LabC mice and four WildR mice at 14- and 56-days post-infection.
