# Supplementary material for: Wild mouse gut microbiota limits initial tuberculosis infection in BALB/c mice
Source: PLoS One. 2023 Jul 26;18(7):e0288290. doi: 10.1371/journal.pone.0288290 (PMC10370681; doi:10.1371/journal.pone.0288290)
Supplement: S2 Fig — Lungs of animals infected for 14, 28 and 56 days were formalin fixed, paraffin-embedded, sectioned and stained with hematoxylin and eosin. Scale bar, 0.5 cm. (PDF) [file pone.0288290.s002.pdf]

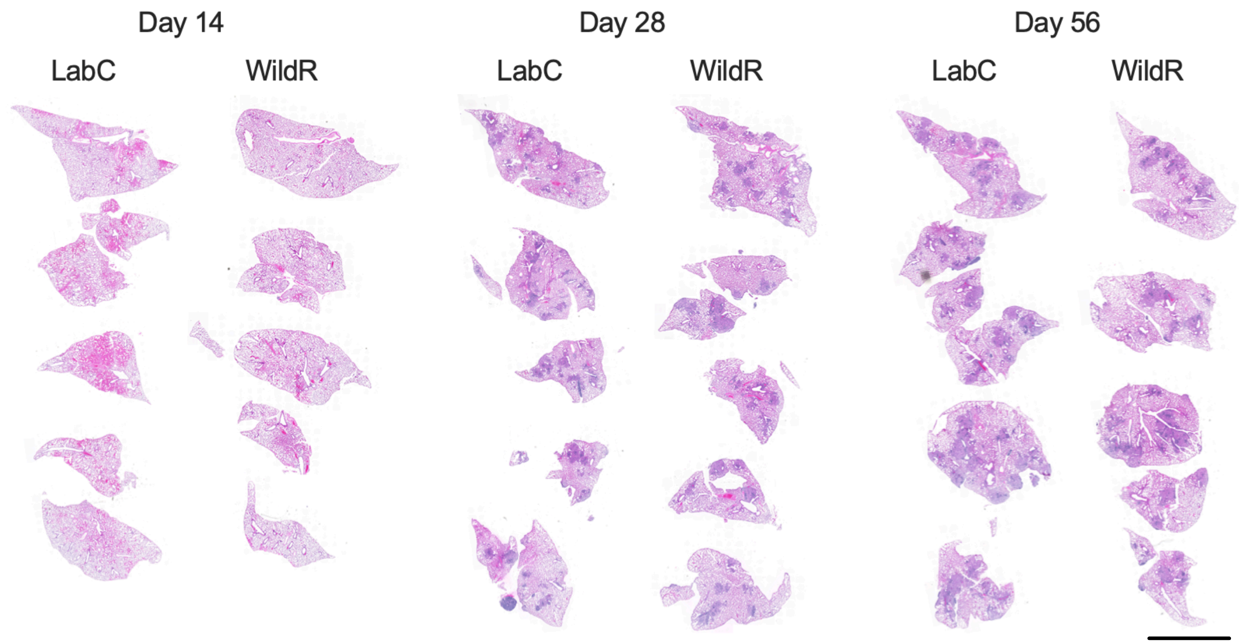


**S2 Figure. Pulmonary TB pathology of LabC mice and WildR.** Lungs of animals infected for 14, 28 and 56 days were formalin fixed, paraffin-embedded, sectioned and stained with hematoxylin and eosin. Scale bar, 0.5 cm.
