# Supplementary material for: Wild mouse gut microbiota limits initial tuberculosis infection in BALB/c mice
Source: PLoS One. 2023 Jul 26;18(7):e0288290. doi: 10.1371/journal.pone.0288290 (PMC10370681; doi:10.1371/journal.pone.0288290)
Supplement: S1 Table — (PDF) [file pone.0288290.s003.pdf]

Animal ID	Day	Read Counts	Animal ID	Day	Read Counts
BALB/c-1	-1	101,017	BALB/c WildR-1	-1	97,613
BALB/c-2	-1	89,027	BALB/c WildR-2	-1	96,079
BALB/c-3	-1	88,528	BALB/c WildR-3	-1	102,124
BALB/c-4	-1	93,088	BALB/c WildR-4	-1	110,126
BALB/c-5	-1	105,717	BALB/c WildR-5	-1	100,788
BALB/c-6	-1	98,567	BALB/c WildR-6	-1	102,078
BALB/c-1	14	89,212	BALB/c WildR-1	14	101,899
BALB/c-2	14	104,031	BALB/c WildR-2	14	99,146
BALB/c-3	14	103,412	BALB/c WildR-3	14	108,807
BALB/c-4	14	88,070	BALB/c WildR-4	14	101,564
BALB/c-5	14	108,023	BALB/c WildR-5	14	92,137
BALB/c-6	14	94,243	BALB/c WildR-6	14	109,152
BALB/c-1	28	108,233	BALB/c WildR-1	28	109,374
BALB/c-2	28	105,194	BALB/c WildR-2	28	111,872
BALB/c-3	28	108,681	BALB/c WildR-3	28	117,110
BALB/c-4	28	101,383	BALB/c WildR-4	28	110,908
BALB/c-5	28	104,127	BALB/c WildR-5	28	112,933
BALB/c-6	28	115,418	BALB/c WildR-6	28	113,867
BALB/c-1	56	102,110	BALB/c WildR-1	56	98,946
BALB/c-2	56	100,413	BALB/c WildR-2	56	106,671
BALB/c-3	56	97,187	BALB/c WildR-3	56	103,096
BALB/c-4	56	94,341	BALB/c WildR-4	56	106,192
BALB/c-5	56	104,474	BALB/c WildR-5	56	101,022
BALB/c-6	56	100,730	BALB/c WildR-6	56	108,322

S1 Table. Read counts of the 16S rRNA V4 region for individual feces samples.
